# RETRACTION: Enhancing Wound Recovery in Chemotherapy‐Induced Leukopenia for Malignant Tumours: A Meta‐Analysis of Acupuncture Treatment Efficacy

**DOI:** 10.1111/iwj.70436

**Published:** 2025-03-26

## Abstract

**RETRACTION:** WangH. , PanJ. , TanZ. , and ZhangM. , “Enhancing Wound Recovery in Chemotherapy‐Induced Leukopenia for Malignant Tumours: A Meta‐Analysis of Acupuncture Treatment Efficacy,” International Wound Journal 21, no. 2 (2024): e14610, 10.1111/iwj.14610.PMC1083043542052913

The above article, published online on 31 January 2024, in Wiley Online Library (http://onlinelibrary.wiley.com/), has been retracted by agreement between the journal Editor in Chief, Professor Keith Harding; and John Wiley & Sons Ltd. Following an investigation by the publisher, all parties have concluded that this article was accepted solely on the basis of a compromised peer review process. The editors have therefore decided to retract the article. The authors did not indicate their agreement with the retraction.

**RETRACTION:** H. Wang , J. Pan , Z. Tan , and M. Zhang , “Enhancing Wound Recovery in Chemotherapy‐Induced Leukopenia for Malignant Tumours: A Meta‐Analysis of Acupuncture Treatment Efficacy,” International Wound Journal 21, no. 2 (2024): e14610, 10.1111/iwj.14610.PMC1083043542052913

The above article, published online on 31 January 2024, in Wiley Online Library (http://onlinelibrary.wiley.com/), has been retracted by agreement between the journal Editor in Chief, Professor Keith Harding; and John Wiley & Sons Ltd. Following an investigation by the publisher, all parties have concluded that this article was accepted solely on the basis of a compromised peer review process. The editors have therefore decided to retract the article. The authors did not indicate their agreement with the retraction.

